# P-1935. Proteomic and transcriptomic signatures of SARS-CoV-2 associated myocarditis

**DOI:** 10.1093/ofid/ofae631.2094

**Published:** 2025-01-29

**Authors:** Simon Pollett, Josh Chenoweth, Clifton Dalgard, Nusrat J Epsi, Stephanie A Richard, Paul W Blair, Allison M Malloy, Catherine Berjohn, Ryan Flanagan, Anuradha Ganesan, David A Lindholm, Katrin Mende, Rhonda E Colombo, Derek Larson, Robert O’Connell, Mark P Simons, David Tribble, Timothy Burgess, Brian Agan, Afshin Beheshti, Mark Haigney

**Affiliations:** Infectious Disease Clinical Research Program, Department of Preventive Medicine and Biostatistics, Uniformed Services University of the Health Sciences, Bethesda, MD, USA, Bethesda, Maryland; Henry M. Jackson Foundation, Bethesda, Maryland; The American Genome Center, Uniformed Services University of the Health Sciences, Bethesda, Maryland; IDCRP HJF, Bethesda, Maryland; Infectious Disease Clinical Research Program, Department of Preventive Medicine and Biostatistics, Uniformed Services University of the Health Sciences, Bethesda, MD, USA, Bethesda, Maryland; Division of Infectious Diseases, Vanderbilt University Medical Center, Nashville, Tennessee; Department of Pediatrics, Uniformed Services University of the Health Sciences, Bethesda, MD, USA, Bethesda, Maryland; Naval Medical Center San Diego, San Diego, California; Tripler Army Medical Center, Honolulu, Hawaii; Infectious Disease Clinical Research Program, USUHS; Henry M. Jackson Foundation for the Advancement of Military Medicine Inc, Bethesda, Maryland; Department of Medicine, Uniformed Services University of the Health Sciences; Brooke Army Medical Center, San Antonio, TX; 1Infectious Disease Clinical Research Program, Department of Preventive Medicine and Biostatistics, Uniformed Services University of the Health Sciences and Brooke Army Medical Center, JBSA Fort Sam Houston, TX, San Antonio, TX; Infectious Disease Clinical Research Program, USUHS; Henry M. Jackson Foundation for the Advancement of Military Medicine, Inc., Bethesda, Maryland; Fort Belvoir Community Hospital and Uniformed Services University, Fort Belvoir, Virginia; Infectious Disease Clinical Research Program, USUHS, Bethesda, Maryland; Infectious Disease Clinical Research Program, Department of Preventive Medicine and Biostatistics, Uniformed Services University of the Health Sciences, Bethesda, MD, USA, Bethesda, Maryland; Infectious Disease Clinical Research Program, Department of Preventive Medicine and Biostatistics, Uniformed Services University of the Health Sciences, Bethesda, MD, USA, Bethesda, Maryland; Infectious Disease Clinical Research Program, Department of Preventive Medicine and Biostatistics, Uniformed Services University of the Health Sciences, Bethesda, MD, USA, Bethesda, Maryland; Infectious Disease Clinical Research Program, Department of Preventive Medicine and Biostatistics, Uniformed Services University of the Health Sciences, Bethesda, MD, USA, Bethesda, Maryland; Stanley Center for Psychiatric Research, Broad Institute of MIT and Harvard, Cambridge, Massachusetts; Military Cardiovascular Outcomes Research, Department of Medicine, Uniformed Services University of the Health Sciences, Bethesda, MD, USA, Bethesda, Maryland

## Abstract

**Background:**

SARS-CoV-2 myocarditis is a rare but serious complication of COVID-19. The host response correlates of SARS-CoV-2 myocarditis remain unclear, thereby limiting therapeutic development. We therefore performed proteomic and transcriptomic phenotyping of SARS-CoV-2 myocarditis in Military Health System (MHS) beneficiaries. We included a bioenergetics focus given the emerging role of mitochondrial function in enteroviral myocarditis.

**Figure:**
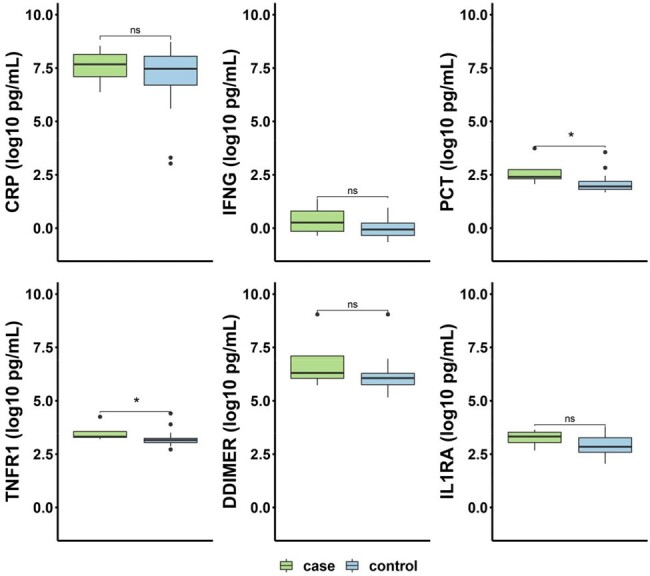

Early (<30 days post infection) plasma proteomic biomarkers measured by the Ella 20-plex ELISA assay in cases (SARS-CoV-2 infections with myocarditis) versus controls (SARS-CoV-2 infections without myocarditis). Values are log10 transformed, and p-values are adjusted for multiple comparisons across the full twenty biomarkers (six shown here; * indicates a p-value < 0.05). CRP = C-Reactive Protein; IFNG = Interferon Gamma ; PCT= Procalcitonin; TNFR1 = Tumor necrosis factor receptor-1; DDIMER = D-dimer; and IL1RA = Interleukin-1 receptor antagonist.

**Methods:**

We previously adjudicated five unvaccinated pre-Delta SARS-CoV-2 myocarditis cases among 5265 MHS beneficiaries who enrolled into the EPICC study with COVID-19; four of these cases had blood collected. We matched myocarditis cases 1:10 with sex, age, comorbidity, and blood sampling-time matched controls (SARS-CoV-2 infection with no myocarditis) from the EPICC study. Cases and controls underwent comparison of early (< 30 days post infection) plasma proteomic biomarkers measured by the Ella 20-plex ELISA assay (with multiplicity adjustment). We also performed early whole blood transcriptomic sequencing with DESeq2 and GSEA analysis of custom mitochondrial pathways, including mitochondrial (mtDNA) regulated innate immune pathways (with FDR for multiple comparisons).

**Figure:**
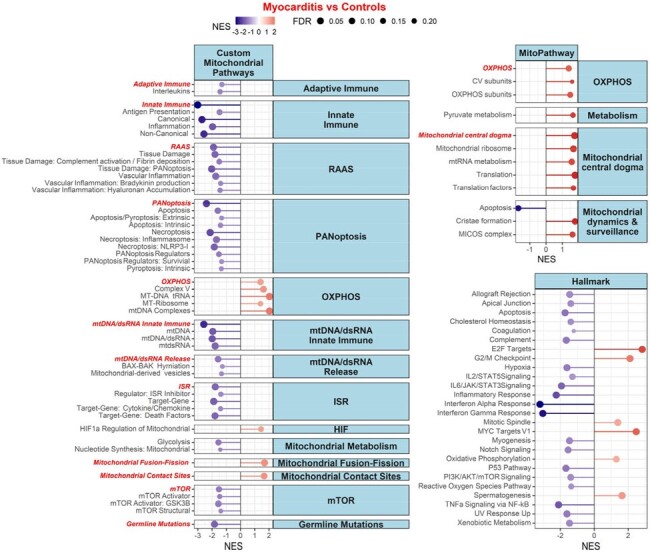

Gene set enrichment analysis of custom mitochondrial pathways in cases (SARS-CoV-2 infections with myocarditis) versus controls (SARS-CoV-2 infections without myocarditis). FDR = False Discovery Rate; NES = Normalized enrichment score.

**Results:**

We detected higher post-infection Tumor Necrosis Factor Receptor-1 (TNFR1) and procalcitonin (PCT) plasma concentrations in myocarditis cases versus matched controls (TNFR1: 0.33 log10 pg/mL difference, p = 0.032; PCT: 0.59 log10 pg/mL difference, p = 0.02) (Fig 1). We noted no statistically significant difference in the 18 other proteomic markers tested. GSEA analysis noted upregulated mitochondrial central dogma and OXPHOS pathway genes, and downregulated mtDNA innate immune genes, in cases versus controls (FDR < 0.05) (Fig 2).

**Conclusion:**

In this MHS study, SARS-CoV-2 myocarditis was associated with elevation of TNFR1 and PCT secretion, upregulated mitochondrial central dogma and OXPHOS gene expression, and downregulated mtDNA innate immunity. While constrained by small sample sizes of a rare complication, these biomarker associations prompt mechanistic studies which may direct development of therapies for myocarditis due to SARS-CoV-2 and perhaps other respiratory viral pathogens.

**Disclosures:**

Simon Pollett, MBBS, AstraZeneca: The IDCRP and HJF were funded to conduct an unrelated phase III COVID-19 monoclonal antibody immunoprophylaxis trial as part of US Govt COVID Response David Tribble, MD, DrPH, AstraZeneca: The IDCRP and HJF were funded to conduct an unrelated phase III COVID-19 monoclonal antibody immunoprophylaxis trial as part of US Govt COVID Response Timothy Burgess, MD, MPH, AstraZeneca: The IDCRP and HJF were funded to conduct an unrelated phase III COVID-19 monoclonal antibody immunoprophylaxis trial as part of US Govt COVID Response

